# Altered AADAC Modulates Trophoblast Invasion and Suggests a Potential Angiogenic Regulatory Role in Severe Preeclampsia

**DOI:** 10.3390/ijms27021103

**Published:** 2026-01-22

**Authors:** Hyo Jung An, Dae Hyun Song, Yu-min Kim, Hyen Chul Jo, Jong Chul Baek, Juseok Yang, Ji Eun Park

**Affiliations:** 1 Department of Pathology, Gyeongsang National University Changwon Hospital, Changwon 51472, Republic of Korea; ariel2020@naver.com (H.J.A.); golgy@hanmail.net (D.H.S.); 2 Department of Pathology, Gyeongsang National University School of Medicine, Jinju 52727, Republic of Korea; 3 Institute of Medical Science, Gyeongsang National University, Jinju 52727, Republic of Korea; kkk_yumin@naver.com (Y.-m.K.); cholida73@naver.com (H.C.J.); gmfather@gnu.ac.kr (J.C.B.); yangandshin@gmail.com (J.Y.); 4 Department of Obstetrics and Gynecology, Gyeongsang National University Changwon Hospital, Changwon 51472, Republic of Korea; 5 Department of Obstetrics and Gynecology, Gyeongsang National University School of Medicine, Jinju 52727, Republic of Korea

**Keywords:** arylacetamide deacetylase, preeclampsia, trophoblast, placenta

## Abstract

Preeclampsia (PE) is a serious pregnancy complication characterized by hypertension and organ dysfunction. Its pathogenesis involves impaired trophoblast invasion and inadequate spiral artery remodeling; however, the underlying molecular mechanisms remain unclear. This study investigated the role of arylacetamide deacetylase (AADAC) in PE and its effects on trophoblast function by analyzing placental tissues from 30 patients with PE and 15 controls. Exploratory RNA sequencing was performed on pooled placental samples from six patients with severe PE and six controls, and AADAC expression was validated by semi-quantitative PCR and Western blotting. HTR8/SVneo cells were exposed to cobalt chloride (CoCl_2_) under hypoxia-mimicking conditions, and AADAC expression was manipulated by siRNA-mediated knockdown (KD) and overexpression (OE). RNA sequencing revealed increased *AADAC* expression in PE placentas (fold change > 2.0, raw *p* < 0.05). Although *AADAC* mRNA levels were elevated in PE tissues, protein levels were reduced. CoCl_2_ exposure was associated with increased expression of AADAC and hypoxia-inducible factor-1 alpha (HIF-1α). Under hypoxia-mimicking conditions, *AADAC* silencing was associated with increased trophoblast invasion and tube formation, whereas *AADAC* overexpression reduced tube formation without significantly affecting invasion. These findings suggest that dysregulated, hypoxia-responsive AADAC expression influences trophoblast invasive and angiogenic behavior in preeclampsia.

## 1. Introduction

Preeclampsia (PE) is a pregnancy-specific disorder characterized by progressive hypertension and endothelial dysfunction that can cause extensive damage to multiple organ systems. It is estimated to occur in 2–8% of all pregnancies and is a major cause of maternal morbidity and mortality worldwide [1,2]. The complications of severe PE can lead to serious maternal health problems, including renal failure, hepatic rupture, seizures (eclampsia), and stroke [3]. Moreover, the only definitive treatment for PE is termination of pregnancy; thus, it often leads to premature birth [4]. Premature birth contributes significantly to perinatal morbidity and mortality and leads to substantial societal costs by increasing childhood morbidity and lifelong disability rates [5].

Recent studies have revealed that PE is a multifactorial syndrome involving complex interactions between impaired trophoblast invasion, maternal vascular maladaptation, immune dysregulation, oxidative stress, and, in some cases, metabolic dysfunction [6,7,8,9]. While the classical two-stage model—characterized by impaired uterine spiral artery remodeling and shallow placental implantation leading to placental ischemia, hypoxia, and the release of antiangiogenic and proinflammatory factors—remains central to our understanding of PE pathogenesis [10,11], additional evidence suggests that metabolic disturbances may also play a role in certain patients. According to one recent study, the serum uric acid/creatinine ratio, which reflects increased uric acid production due to xanthine oxidase overactivity, can serve as a useful marker for predicting the onset and severity of PE as well as adverse maternal and neonatal outcomes [12]. Although this suggests the importance of metabolic markers in disease prediction and pathogenesis, this process is not fully understood, indicating the need for further investigations into the biological behaviors of trophoblasts and their roles in invasion and angiogenesis [13,14].

Given the incomplete understanding of the precise etiology of PE, recent studies have increasingly employed next-generation sequencing (NGS) to identify molecular pathways and pivotal genes associated with its pathogenesis [15]. In alignment with this trend, our NGS analysis of placental tissues from severe PE identified several candidate genes, among which arylacetamide deacetylase (*AADAC*) emerged as a notable candidate. Previous reports have suggested AADAC’s potential relevance to placental biology. For example, Chang et al. [16] observed higher AADAC expression in placentas from patients with PE alone than in those with superimposed PE, and Dupressoir et al. [17] demonstrated that altered AADAC expression accompanies syncytiotrophoblast defects and intrauterine growth restriction in syncytin-B knockout mice. While these findings imply that AADAC may influence placental structure or function, its specific role in PE remains unclear.

AADAC is a microsomal enzyme primarily involved in lipid metabolism and oxidative processes [18]. Successful trophoblast invasion and spiral artery remodeling are thought to involve dynamic cellular membrane reorganization and increased metabolic demand, processes that may depend in part on lipid homeostasis. A previous study reported gestation-dependent changes in placental lipid content and lipid metabolic enzyme abundance, indicating that lipid metabolic pathways are dynamically regulated during placental development [19]. However, the potential involvement of AADAC-mediated lipid metabolic regulation in trophoblast function, particularly under hypoxic stress conditions characteristic of PE, has not been investigated.

Based on these observations, we hypothesized that altered AADAC expression under hypoxia-mimicking conditions may be associated with changes in trophoblast invasion and angiogenic behavior. Accordingly, this study was designed to investigate the potential role of AADAC in trophoblast function and its possible involvement in the development of PE.

## 2. Results

### 2.1. Identification of Three Upregulated Protein-Coding Genes in Human Placental Cells Subjected to Severe Preeclampsia

**Figure 1:**
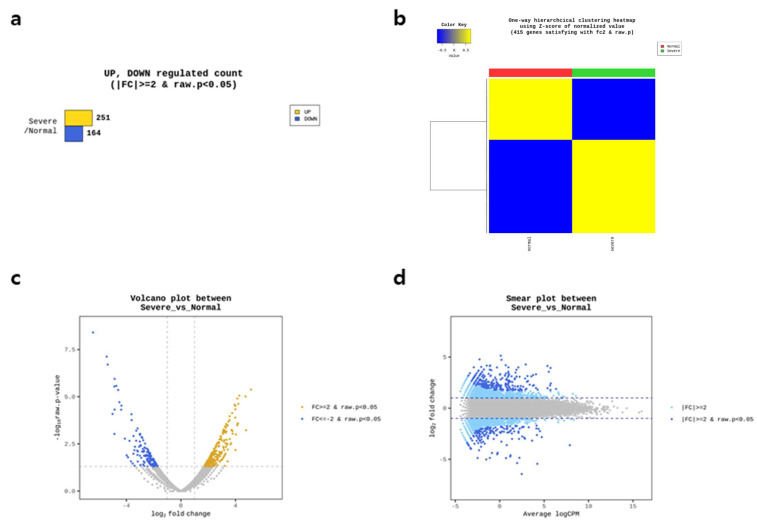
Identification of pivotal protein coding genes in severe preeclampsia (PE) (**a**) Upregulated and downregulated genes of severe PE. (**b**) One-way hierarchical clustering heatmap using Z score for normalizing values. (**c**) Volcano plot used to evaluate log2-fold change and *p* value obtained from comparison of two groups (X-axis: log2-fold fold change, Y-axis: − log10 *p* value). (**d**) Smear plot drawn on basis of overall average expression level to confirm genes that presented greater expression differences in severe PE group (X-axis: average logCP; Y-axis: log2 fold change).

To elucidate the pivotal genes involved in the pathogenesis of PE, we used mRNA sequencing and detected genes associated with severe PE. Among the 415 protein-coding genes whose expression differed significantly from that of the control group (fold change > 2 or < −2, raw *p* < 0.05), 251 were upregulated and 164 were downregulated in the severe PE group (Figure 1a). The one-way hierarchical clustering heatmap revealed two clusters of upregulated and downregulated protein-coding genes between the severe PE and control groups (Figure 1b). The upregulated protein-coding genes are shown in the right and upper regions of the volcano and smear plots, respectively, whereas the downregulated protein-coding genes are shown in the left and lower regions of the volcano and smear plots, respectively (Figure 1c–d).

**Figure 2:**
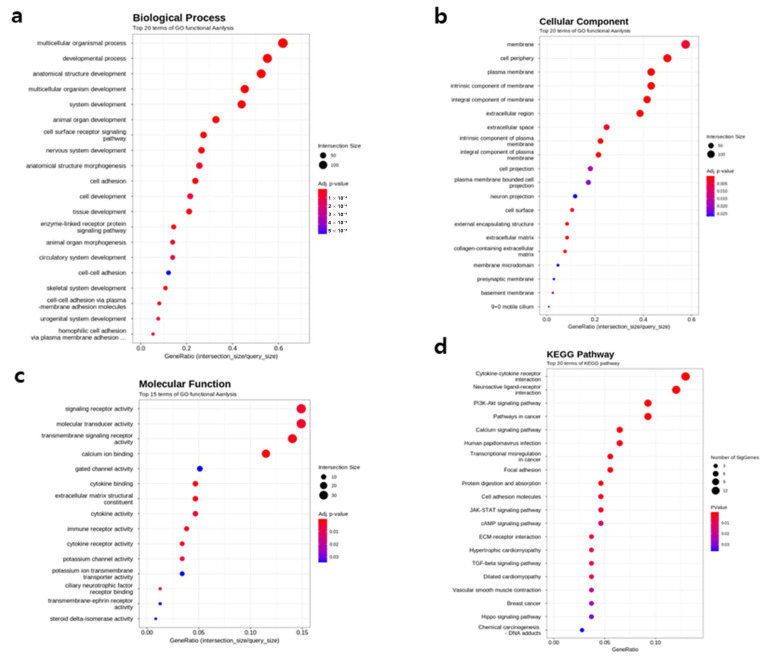
Gene Ontology (GO) enrichment and KEGG pathway of severe PE. Biological process (**a**), cellular component (**b**), and molecular function (**c**) enrichment analysis results. The top 20 enriched KEGG pathways (**d**) including the PI3k-Akt pathway, the TGF-beta pathway, the Hippo signaling pathway, and vascular smooth muscle contraction pathways.

The GO enrichment analysis using g:Profiler revealed that the upregulated genes in severe PE were mainly involved in biological processes such as cell adhesion, extracellular matrix organization, development, and lipid metabolism and were localized to the plasma membrane and extracellular region, showing enrichment of signaling receptor activity (Figure 2a–c). The top 20 upregulated protein-coding genes, ranked by fold change, are listed in Supplementary Table S1. The top 20 KEGG pathways included the PI3K–Akt, TGF-beta, and Hippo signaling pathways as well as the vascular smooth muscle contraction pathway (Figure 2d). Among the upregulated genes, GO terms such as anatomical structure development, cell adhesion, and vascular development were enriched. *LAIR2* and *MMRN1* were primarily associated with immune and vascular development–related categories, whereas *AADAC* was linked to metabolic and developmental processes. These results suggest that *LAIR2*, *MMRN1*, and *AADAC* collectively participate in pathways regulating trophoblast differentiation and vascular morphogenesis. Based on fold-change magnitude, *LAIR2, MMRN1,* and *AADAC* were among the most upregulated genes in severe PE placentas compared with controls. Therefore, they were selected for further analysis due to their potential roles in immune regulation, angiogenesis, and lipid metabolism, respectively.

### 2.2. Higher Relative mRNA Expression Levels of AADAC and Lower Relative Protein Expression Levels in Trophoblasts Subjected to Severe PE

**Figure 3:**
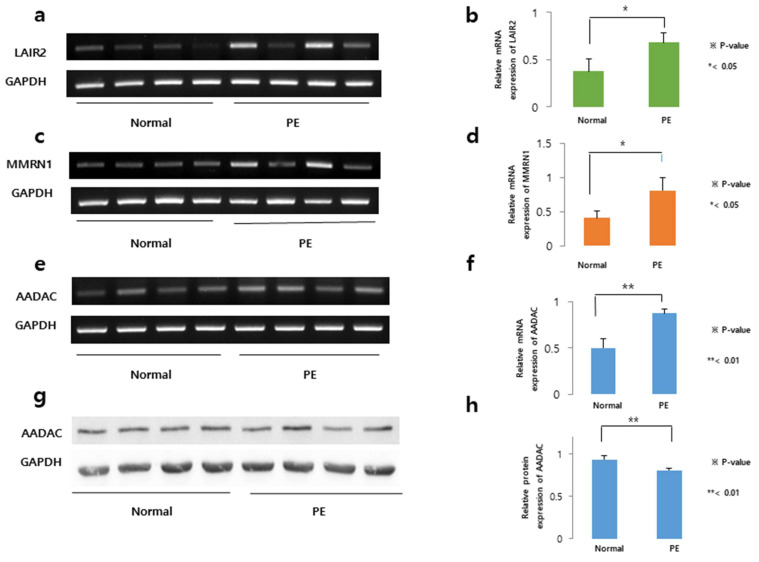
Upregulated genes in human trophoblast cells. The mRNA expression levels of *LAIR2* (**a**), *MMRN1* (**c**), and arylacetamide deacetylase (*AADAC*) (**e**) were evaluated in four normal and four severe PE human placental tissues. The relative mRNA expression levels of *LAIR2* (**b**), *MMRN1* (**d**), and *AADAC* (**f**) were significantly higher in severe PE placental cells than in control placental cells. (**g**) The protein expression levels of AADAC were evaluated in four normal and four severe PE human placental samples. (**h**) The relative protein expression of AADAC was significantly lower in severe PE placental tissues than in control placental tissues. The relative mRNA and protein expression levels were normalized to an internal control, GAPDH. Two-tailed Student’s *t* tests were used for comparisons between two groups. Original uncropped Western blot images corresponding to this figure are provided as non-published materials for editorial review.

The mRNA expression levels of *LAIR2*, *MMRN1*, and *AADAC* were evaluated in independent placental tissue samples (*n* = 4 per group) randomly selected from women with normal deliveries and patients with severe PE. The relative mRNA expression of *LAIR2* was significantly higher in the placental tissues subjected to severe PE (0.7) than in control placental tissues (0.4) (*p* < 0.05) (Figure 3a,b). Similarly, for *MMRN1*, the relative mRNA expression was 0.8 in the severe PE group and 0.4 in the control group, with a statistically significant difference (*p* < 0.01) (Figure 3c,d). For *AADAC*, the value was 0.9 in the severe PE group and 0.5 in the control group, also with a significant difference (*p* < 0.01) (Figure 3e,f). Among the three genes investigated, *AADAC* showed the most significant differential expression between placental cells in the severe PE and control groups. To determine whether this transcriptional upregulation translated to protein-level changes, we evaluated AADAC protein expression in human placental tissues from four women with normal pregnancies and four patients with severe PE, the same samples described above used for the determination of mRNA expression levels. Interestingly, while *AADAC* mRNA expression showed an upward trend, its relative protein level appeared slightly lower in severe PE placentas (*p* < 0.01) (Figure 3g,h). This inverse relationship between the mRNA and protein levels highlights the complex regulation of AADAC in the pathophysiology of PE.

### 2.3. Higher AADAC Expression Levels in PE Placental Cells After 18 h of Hypoxia

**Figure 4:**
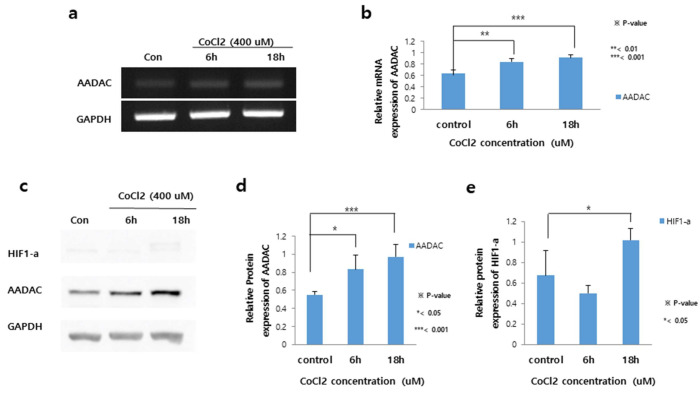
AADAC expression levels after hypoxic treatment. (**a**,**b**) HTR8/SVneo cells cultured with 400 µM cobalt chloride (CoCl_2_) for both 6 h and 18 h presented significantly higher AADAC mRNA expression than control cells (** *p* < 0.01 and *** *p* < 0.001, respectively). (**c**,**d**) HTR8/SVneo cells cultured with 400 µM CoCl_2_ for both 6 h and 18 h presented significantly higher *AADAC* protein expression than control cells (* *p* < 0.05 and *** *p* < 0.001, respectively). (**e**) HTR8/SVneo cells cultured with 400 µM CoCl_2_ for 18 h presented significantly higher hypoxia-inducible factor-1 alpha (HIF1α) protein expression than control cells (* *p* < 0.05). Two-tailed Student’s *t* tests were used for comparisons between two groups. Original uncropped Western blot images corresponding to this figure are provided as non-published materials for editorial review.

AADAC showed the most significant differential expression between severe PE placental cells and control placental cells. Thus, we further evaluated AADAC mRNA and protein expression and performed functional tests of its function in HTR8/SVneo trophoblasts. In addition, we hypothesized that hypoxia might induce the development of a PE-like status in HTR8/SVneo trophoblasts, which are normal placental cells. We used CoCl_2_ as a hypoxia-mimicking agent. Compared with control cells, HTR8/SVneo cells cultured with 400 µM CoCl_2_ for both 6 h and 18 h presented significantly higher *AADAC* mRNA expression (*p* < 0.01 and *p* < 0.001, respectively) (Figure 4a,b). In addition, HTR8/SVneo cells cultured with 400 µM CoCl_2_ for both 6 h and 18 h presented significantly higher *AADAC* protein expression than did the control cells (*p* < 0.05 and *p* < 0.001, respectively) (Figure 4c,d). However, compared with control cells, HTR8/SVneo cells cultured with 400 µM CoCl_2_ only presented significantly higher HIF1α protein expression at the 18 h time point (*p* < 0.05) (Figure 4c,e).

The role of HIF1α in the pathogenesis of PE has been established in several studies; the findings suggest that HIF1α expression levels are increased not only in early-gestation placentas in low-oxygen environments but also in PE placentas, during shallow trophoblast invasion with consequent placental hypoxia [20,21,22,23]. As previous studies have suggested that hypoxia suppresses the invasion and migration of trophoblasts by inhibiting the SRC-3/AKT/mTOR pathway [24], we further performed tube formation experiments. In terms of tube formation, hypoxia-treated cells presented significantly fewer meshes than did the control cells after both 6 h and 18 h (40 and 20 meshes vs. 70 meshes, *p* < 0.001 and *p* < 0.0005, respectively) (Figure S2).

### 2.4. Higher Numbers of Meshes in Tube Formation as Well as Invading Cells After AADAC Silencing and 18 h of Hypoxia

**Figure 5:**
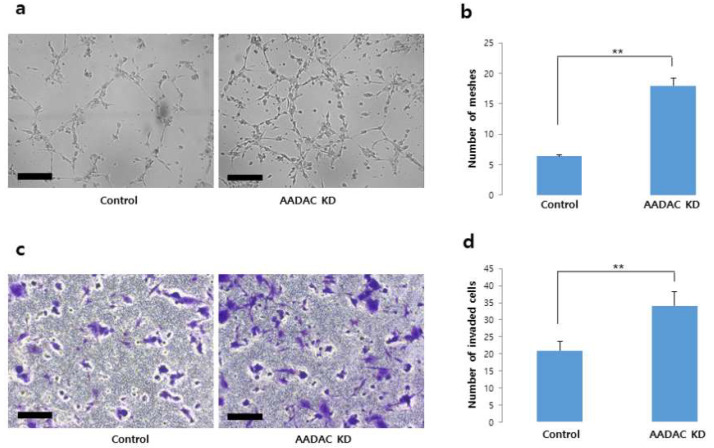
Tube formation and invasion assays in *AADAC*-KD HTR8/SVneo trophoblasts. (**a**,**b**) *AADAC*-KD cells exposed to hypoxia (400 µM CoCl_2_, 18 h) formed a significantly higher number of meshes and junctions compared with control cells, indicating enhanced angiogenic potential (×40; scale bar = 100 µm). (**c**,**d**) The number of invading *AADAC*-KD cells after 18 h of hypoxic treatment was also significantly higher than that of control cells (×40; scale bar = 100 µm). Data are presented as mean ± SEM from three independent experiments. Statistical significance is indicated as ** *p* < 0.01. Data represent relative mRNA or protein expression levels normalized to GAPDH.

Since trophoblasts cultured for 18 h presented more significant differences in *AADAC* mRNA and protein expression and HIF1α protein expression levels as well as fewer meshes during tube formation, we performed 18 h rather than 6 h of hypoxia and *AADAC*-KD cell experiments, including tube formation and invasion assays. Compared with control cells, *AADAC*-KD HTR8/SVneo cells cultured with 400 µM CoCl_2_ for 18 h presented significantly lower *AADAC* mRNA expression (*p* < 0.001). However, AADAC protein expression did not differ significantly between the two cell lines (Figure S3). In terms of tube formation, after 18 h of hypoxia, *AADAC*-KD cells presented a significantly higher number of meshes (19) than did control cells (7) (*p* < 0.01) (Figure 5a,b). In the cell invasion test, after 18 h under hypoxia-mimicking conditions, the number of invading cells was significantly higher in the *AADAC*-KD group (35) than in the control group (20) (*p* < 0.01) (Figure 5c,d). These findings suggest that cell recovery after *AADAC* KD promotes invasion and tube formation in hypoxia-treated or damaged HTR8/SVneo trophoblasts.

### 2.5. Decreased Number of Meshes but Not Trophoblast Invasion Due to AADAC Overexpression

**Figure 6:**
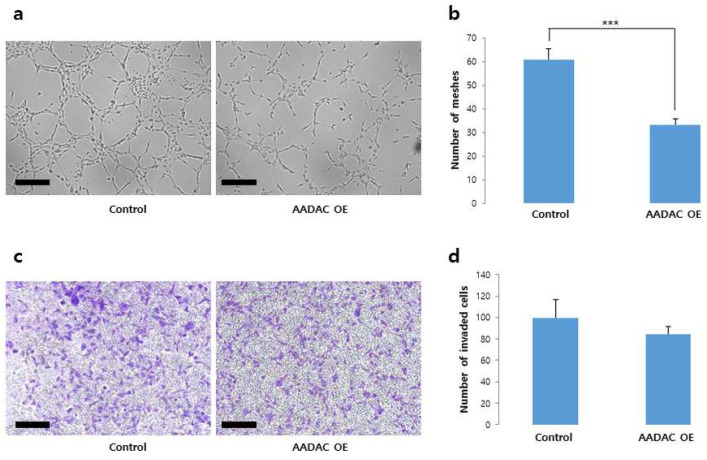
Tube formation and invasion assays in AADAC-OE HTR8/SVneo trophoblasts. (**a**,**b**) *AADAC*-OE cells showed a markedly reduced number of tubular meshes compared with control cells, suggesting decreased angiogenic activity (×40; scale bar = 100 µm; *** *p* < 0.001). (**c**,**d**) The number of invading *AADAC*-OE cells was lower than that of control cells, although the difference was not statistically significant (×40; scale bar = 100 µm). Data are presented as mean ± SEM from three independent experiments.

To determine whether *AADAC* OE alone is sufficient to replicate the effects of hypoxia or mimic the characteristics of PE, we generated *AADAC*-OE trophoblasts for further study. Analysis of *AADAC* mRNA expression revealed a significant increase in *AADAC*-OE HTR8/SVneo cells compared with control cells (*p* < 0.001). However, *AADAC* protein expression did not differ significantly between the two groups (Figure S4). In terms of tube formation, *AADAC*-OE cells presented a significantly smaller number of meshes (30) than did control cells (60) (*p* < 0.001) (Figure 6a,b). In the cell invasion test, the number of invaded cells was lower in the *AADAC*-OE group (90) than in the control group (100), but the difference was not statistically significant (Figure 6c,d). After 18 h of hypoxia-mimicking conditions, unlike *AADAC* silencing, *AADAC* OE resulted in fewer meshes in tube formation than did the control. In addition, no significant difference was observed in the invasion assays of *AADAC*-OE cells.

## 3. Discussion

Our study, aimed at elucidating the role of AADAC in the pathogenesis of PE, provides the first evidence that *AADAC* is present in human placental trophoblasts and is significantly differentially expressed in patients with PE. Exposure to CoCl_2_ under hypoxia-mimicking conditions significantly upregulated AADAC mRNA and protein expression in HTR8/SVneo cells. Furthermore, AADAC silencing promoted invasion and tube formation in HTR-8/SVneo cells under hypoxia-mimicking conditions, whereas AADAC OE impaired tube formation but did not significantly decrease cell invasion. These findings indicate that AADAC plays a regulatory role in trophoblast invasion and angiogenic behavior under hypoxia-mimicking conditions, providing new insights into the molecular pathways contributing to PE pathogenesis.

Defective trophoblast invasion and poor spiral artery remodeling are fundamental features of PE development [10], and the primary causes are likely abnormal trophoblast invasion and vascular formation caused by the hypoxic state. Given that the biological characteristics of trophoblasts are essential for normal placental development, it is important to determine whether differential placental AADAC expression in PE may contribute to abnormal trophoblast function. Currently, trophoblast cell biology research is primarily conducted using the human extravillous trophoblast HTR-8/SVneo cell line, and hypoxic culture of trophoblasts in vitro is a commonly used model to simulate PE in vitro [24]

AADAC is essential for lipid metabolism and promotes triglyceride hydrolysis in the gastrointestinal tract, and its regulation of adipose tissue and liver functions is linked to diseases such as obesity and liver disorders [18]. While investigating AADAC in cancer, Wang et al. [25] observed upregulated expression in ovarian cancer tissues. Interestingly, AADAC OE in ovarian cancer cell lines was found to attenuate cell proliferation, migration, and invasion while also synergistically enhancing the tumor-suppressive effects of chemotherapeutic agents. In a separate study, Toyohara et al. [26] found increased *AADAC* in the vascular smooth muscle cells of type 2 diabetic patients protected from CVD, suggesting a role in vascular remodeling. In the placenta, such lipid-metabolic and vascular remodeling functions of *AADAC* may influence trophoblast behavior and contribute to placental vascular adaptation, both of which are impaired in PE. Our findings extend these insights to placental biology, suggesting that *AADAC* influences trophoblast invasion and angiogenic potential under hypoxia-mimicking conditions, processes that are critical for maintaining placental oxygenation and may become dysregulated in PE.

Notably, several signaling pathways are involved in trophoblast invasion and angiogenesis. In a mouse model, Notch signaling activity was found to be highest in endovascular placental trophoblasts, and conditional depletion of Notch2 markedly reduced maternal blood canal size and placental perfusion, leading to embryonic death [27]. Notch signaling has also been shown to activate downstream effectors of the PI3K/Akt pathway [28], which may functionally link these pathways in regulating vascular remodeling. In our study, KEGG pathway analysis revealed that the PI3K/Akt pathway was significantly altered in severe PE placentas, consistent with its established role—together with its downstream effector mTOR—in regulating trophoblast invasion and migration under hypoxic conditions [24,29,30]. Although a direct connection between AADAC and the PI3K/Akt/mTOR pathway has not yet been established, changes in AADAC expression could indirectly modulate these signaling cascades and contribute to the dysregulated trophoblast behavior observed in PE. Further investigations are warranted to clarify this relationship. Overall, these findings support the idea that AADAC may participate in the complex network of pathways controlling trophoblast invasion and angiogenesis in the pathogenesis of PE. Importantly, the present study does not provide direct experimental evidence linking AADAC to specific angiogenic or intracellular signaling pathways. The pathway associations discussed above are derived from transcriptomic pathway enrichment analysis and functional phenotypic observations, and therefore should be interpreted as hypothesis-generating observations rather than definitive mechanistic conclusions.

The three genes selected for validation—LAIR2, MMRN1, and AADAC—have biological functions potentially related to placental development and PE pathogenesis. LAIR2 encodes a soluble inhibitory receptor that modulates immune activation by competing with LAIR1 for collagen binding, thereby contributing to immune regulation at the maternal–fetal interface [31,32]. MMRN1 is an extracellular matrix protein expressed in platelets and endothelial cells and has been reported to influence endothelial adhesion and angiogenic processes essential for vascular remodeling in the placenta [33]. AADAC, as discussed above, is implicated in lipid metabolic and oxidative processes within the placenta. Together, these genes represent distinct biological pathways: immune modulation, angiogenesis, and lipid metabolism, which may collectively contribute to the impaired trophoblast function and vascular dysregulation observed in PE. Further studies are warranted to examine whether AADAC influences the expression of key proand anti-angiogenic mediators, which would help clarify its potential role in placental vascular remodeling.

Interestingly, in vivo, the mRNA expression levels of LAIR2, MMRN1, and AADAC were increased in PE placentas, but only AADAC protein expression was significantly lower than in controls. This discrepancy between mRNA and protein expression suggests that multiple layers of regulatory control may be involved in AADAC expression:

(1) Posttranscriptional regulation: Systematic analyses across human tissues have shown that mRNA–protein correlations vary considerably and that protein abundance is often determined at the translational rather than transcriptional level during cellular stress [34]. In the placenta, hypoxic stress—a key feature of PE—can induce such posttranscriptional mechanisms, including changes in mRNA stability, miRNA-mediated repression, and reduced translation efficiency [35,36]. Thus, the mismatched AADAC mRNA and protein expression pattern might indicate that stress suppresses translation.
(2) Posttranslational modifications (PTMs) and protein turnover: Increased protein degradation or decreased stability could also explain the reduced AADAC protein levels despite elevated mRNA levels [37]. Abnormal PTMs can alter protein conformation, localization, or antibody recognition, ultimately impairing function [38]. PE-associated oxidative stress may trigger such PTMs or proteasomal degradation events affecting AADAC stability. Additionally, protein activity often depends on subcellular localization and interactions rather than abundance alone [39].
(3) Compensatory and signaling mechanisms: Alternative signaling pathways or isoform variations may also influence AADAC expression. Cellular stress and signaling can affect the splicing machinery, producing distinct isoforms that modify translation efficiency and function. Such mechanisms may underlie compensatory adjustments in AADAC regulation [40]. Taken together, these findings suggest that multiple regulatory layers, including posttranscriptional control, PTMs, and compensatory signaling, may collectively account for the discordant AADAC mRNA and protein expression observed in PE placentas. Notably, while AADAC OE significantly altered tube formation, it did not affect invasion, in contrast to the effects observed following AADAC KD. This functional asymmetry suggests that AADAC may be more closely associated with angiogenic behavior and blood vessel–like structure formation than with trophoblast invasion. Such divergent effects between knockdown and overexpression conditions may reflect threshold-dependent or localization-dependent regulation, rather than a simple linear relationship between AADAC expression levels and cellular function. In this context, modest changes in AADAC expression may be sufficient to influence angiogenic properties, whereas further increases beyond a certain level may not proportionally affect invasive behavior. These findings are consistent with prior reports linking AADAC to cell migration and proliferation [26], and further suggest that AADAC may exert context-dependent functional roles within the placental microenvironment.

Despite its key findings, our study has several limitations that should be carefully considered during their interpretation. First, the small clinical sample size limits statistical power and generalizability. Second, notable differences in maternal BMI, weight, and gestational age were observed between the control and PE groups, which may have acted as potential confounding factors influencing placental gene expression. Third, our in vitro experiments using HTR8/SVneo cells under a CoCl_2_-induced hypoxia-mimicking model provide only a simplified approximation of the complex placental environment observed in PE. Although CoCl_2_-induced chemical hypoxia and the immortalized nature of the HTR8/SVneo cell line may not fully reproduce the physiological behavior of primary trophoblasts, this approach is commonly used due to its reproducibility and responsiveness to hypoxic stress. Fourth, the RNA-sequencing analysis was performed using pooled samples and was therefore designed as an exploratory screening approach. Accordingly, the transcriptomic findings should be interpreted as hypothesis-generating rather than confirmatory. Fifth, a discrepancy between AADAC mRNA and protein levels was observed, suggesting regulatory mechanisms not fully explored in this study. In addition, gene expression validation relied on semiquantitative PCR rather than RT-qPCR; the inherent methodological limitations, particularly under hypoxic conditions, may restrict quantitative precision. As a result, these data were interpreted as indicative of general directional trends rather than definitive quantitative measurements. Sixth, the functional assays relied on manual cell counting without a formally blinded protocol, which may introduce observer-related variability. In addition, the absence of specific proliferation or viability assays limits the interpretation of functional readouts. Finally, while an association between AADAC and trophoblast invasion and tube formation was established, the specific angiogenic or intracellular signaling pathways involved were not directly examined and therefore remain unclear. Future studies using larger, more closely matched cohorts and animal models are warranted to confirm these findings and delineate how AADAC contributes to PE pathophysiology.

## 4. Materials and Methods

To investigate the potential function of AADAC in PE, we conducted transcriptomic analysis of placental tissues followed by functional validation using an in vitro trophoblast model.

### 4.1. Patient Samples

Placental tissue samples were collected from women who delivered at Gyeongsang National University Changwon Hospital between February 2021 and January 2023. The study included 30 patients with PE and 15 normotensive controls who all underwent cesarean delivery. Participants’ clinical characteristics are summarized in Table 1.

Among these, placental samples from six women with severe PE and six matched controls were used for RNA sequencing. Independent samples (n = 4 per group) were subsequently used for validation experiments, including semiquantitative PCR and Western blot analyses. PE and severe PE were diagnosed in accordance with the American College of Obstetrics and Gynecology criteria [3]. All deliveries were singletons, and patients with diabetes, cardiovascular disease, chronic kidney disease, chronic hypertension, or metabolic disease were excluded.

### 4.2. Trophoblast Isolation

Primary human trophoblasts were isolated using Percoll gradients [41]. Villous tissue was separated from vessels and connective tissue, minced, and washed with DPBS before being digested three times at 37 °C for 20 min in enzyme medium containing 1 mg/mL Dispase II, 0.5 mg/mL collagenase I, and 0.1 mg/mL DNase I. The digested tissue was subsequently neutralized with complete DMEM, filtered through 100-μm and 40-μm strainers, and centrifuged at 350× *g* for 10 min at 4 °C. Cell suspensions were layered on a Percoll gradient (65%, 55%, 50%, 45%, 35%, 30%, and 25%) and centrifuged at 730× *g* for 30 min at 4 °C to isolate trophoblasts. The 45–35% Percoll layer containing trophoblasts was collected, washed, and centrifuged at 350× *g* for 10 min at 4 °C. The isolated cells were then cultured in DMEM (Gibco, #11995-065) supplemented with 10% fetal bovine serum (FBS, GenDEPOT, #F0900), 1% penicillin–streptomycin (Corning, #30-002-CI), and Primocin (Invitrogen, ant-pm-2) at 37 °C in 5% CO_2_. To ensure the purity of the isolated cell population, cell blocks were prepared immediately after isolation and compared with tissue slides prepared from the chorionic disc of the placenta. The cell blocks exhibited a monotonous population consisting of a single cell type with abundant cytoplasm and an epithelioid morphology (Figure S1). Based on these characteristics, the isolated cells were most consistent with trophoblasts among the cell types found in the chorionic disc. The purity of isolated trophoblast cells was evaluated by immunohistochemical staining for cytokeratin 7 (CK7) and CD31. Most of the cells showed cytoplasmic CK7 positivity, whereas all cells were negative for CD31, confirming minimal endothelial contamination. Thus, subsequent experiments were performed using these verified trophoblast cells. The placental tissues were processed immediately after collection and flash frozen at −80 °C within 1 h of surgery.

### 4.3. RNA Extraction and Next-Generation Sequencing Analysis

RNA samples from six normal placental samples and six severe PE placental samples were separately extracted and pooled into two groups for RNA sequencing. Library preparation and validation quality checks were performed before NGS. Ribodepletion was conducted using Ribo-Zero H/M/R Gold. Employing an Agilent Technologies 2100 Bioanalyzer, the total RNA integrity was checked, and samples with an RNA integrity number of at least 7 were retained. The template size distribution was checked on an Agilent Technologies 2100 Bioanalyzer using a DNA 1000 chip to verify the size of the PCR-enriched fragments. Transcriptome sequencing was performed with the Illumina platform. The raw RNA sequencing data were extracted as fragments per kilobase of exons per million fragments mapped values for each sample. Candidate genes were identified using edgeR (version 3.26.8), which employs statistical methods based on the negative binomial distribution, including empirical Bayes estimation, exact tests, generalized linear models, and quasi-likelihood tests. The fold change was defined as the ratio of normalized expression values (severe PE/control), and the greatest fold change represented the most extreme ratio observed between the two conditions. Cutoffs of |fold change| ≥ 2 and raw *p* < 0.05 (exact test) were used to define significance. Gene Ontology (GO) enrichment analysis was performed using g:Profiler, which maps genes to biological categories based on GO terms and Entrez Gene identifiers. Pathway enrichment was assessed using the KEGG database (version v20230102) to identify significantly enriched molecular pathways. Due to the pooled-sample design, this RNA-sequencing analysis was conducted as an exploratory screening to identify candidate genes rather than to define definitive differentially expressed genes; therefore, raw *p*-values were used without false discovery rate (FDR) correction.

### 4.4. Semiquantitative PCR

Total RNA was extracted from human placental cells using TRIzol reagent (Qiagen, Hilden, Germany) and quantified using the QIAxpert System (Qiagen). After reverse transcribing 1 µg of total RNA to cDNA using the Maxime RT PreMix Kit (iNtRON, #25081, Burlington, MA, USA), equal amounts of synthesized cDNA (1 µg) were subjected to semiquantitative PCR via the Maxime PCR PreMix Kit (iNtRON, #25025) at 58 °C with 40 cycles of PCR runs. The primers used in these experiments were as follows: LAIR2 forward, 5′-GGGAGCCATGTGACTTTCAT-3′ and reverse, 5′-TCACTGTGCTCAGACCATCC-3′; MMRN1 forward, 5′-TGGAGATCCTTCAACCCTTG-3′ and reverse, 5′-TTGAGGCCATCTTCCATTTC-3′; GAPDH forward, 5′-GTCCACCACCCTGTTGCTGTAG-3′; reverse, 5′-CAAGGTCATCCATGACAACTTTG-3′. The *AADAC* (Bioneer, Daejeon, South Korea; #P290849) primers were purchased from Bioneer. The relative mRNA expression levels were normalized to that of GAPDH, which was used as an internal control. Semiquantitative PCR was performed under carefully optimized, non-saturating conditions using independent biological replicates (n = 4 per group). Band intensities were analyzed by densitometry and statistically compared to assess expression differences.

### 4.5. Western Blot Analysis

Proteins were extracted from HTR8/SVneo cells using RIPA lysis buffer (Thermo Fisher Scientific, Waltham, MA, USA; #89900) containing a protease inhibitor cocktail (#78430). Equal amounts of protein (40 µg) were separated by SDS–PAGE (10–12% gels; 45 kDa for AADAC, 120 kDa for hypoxia-inducible factor-1 alpha (HIF1α), and 37 kDa for GAPDH) and transferred onto 0.45-µm nitrocellulose membranes. After blocking with 5% non-fat dry milk in TBST for 1 h, membranes were incubated overnight at 4 °C with primary antibodies (anti-AADAC (1:500, Biorbyt, Cambridge, UK; #orb560902), anti-HIF-1α (1:500, Abcam, #ab51608), and anti-GAPDH (1:1000, Abcam, Cambridge, UK; #ab8245)) followed by HRP-conjugated secondary antibodies (1:5000, Jackson ImmunoResearch, West Grove, PA, USA) for 1 h. Protein bands were visualized using enhanced chemiluminescence (Thermo Fisher Scientific, #32109) and captured on a Fusion Solo System (Vilber, Marne-la-Vallée, France). Band intensities were quantified in ImageJ (V.1.53K; Wayne Rasband; National Institutes of Health, Bethesda, MD, USA) and normalized to GAPDH. Relative expression values are shown as mean ± SEM from three independent experiments.

### 4.6. Cell Lines and Reagents

HTR8/SVneo human placental cells were purchased from the Korean Cell Line Bank (Seoul, Republic of Korea) and cultured in RPMI 1640 (Gibco, Thermo Fisher Scientific, Waltham, MA, USA; #11875-093) supplemented with 10% FBS (Gibco, #26140-079) and 1% penicillin–streptomycin (Corning, NY, USA;#30-002-CI) at 37 °C in an atmosphere containing 5% CO_2_.

### 4.7. Hypoxia

CoCl_2_ was used as a hypoxia-mimicking agent [42]. To determine the appropriate amount of CoCl_2_, 2 × 10^6^ HTR8/SVneo cells/mL were seeded in 10 cm diameter dishes. For the experiments, HTR8/SVneo cells were cultured in the absence or presence of 400 µM CoCl_2_ for 6 or 18 h.

### 4.8. AADAC Knockdown

HTR8/SVneo cells were plated at a density of 8.2 × 10^5^ cells/well in 12-well plates and transiently transfected with human *AADAC* siRNA (Bioneer) or negative control scrambled siRNA (Bioneer, #SN-1002) at a final concentration of 50 nM via Lipofectamine 3000 (Invitrogen, Thermo Fisher Scientific, Waltham, MA, USA; #L3000015). The siRNA sequence used was as follows: *AADAC*, 5-AAGTATCATTTCCCAATTCAATT-3. The cells were incubated for 72 h before harvesting. The experiments were repeated independently three times.

### 4.9. Generation of AADAC-Overexpressing Stable Cell Lines

HTR8/SVneo cells were transfected with the pCMV3-*AADAC* plasmid (Sino Biological Inc., Beijing, China; #HG10639-ACG). At 48 h after transfection and every 2–3 d thereafter, the cells were cultured with fresh RPMI containing hygromycin B (Sigma, St. Louis, MO, USA) at a final concentration of 250 µg/mL. Parental HTR8/SVneo cells obtained from the American Type Culture Collection (ATCC, Manassas, VA, USA; CRL-3271) were used as transfection controls.

### 4.10. Cell Invasion Assay

Cell invasion was assessed using a 24-well cell invasion assay kit (CytoSelect, Cell Biolabs, Inc., San Diego, CA, USA; #CBA-110) according to the manufacturer’s instructions. HTR8/SVneo cells were seeded at 2.5 × 10^4^ cells/mL in the upper transwell chamber. The number of invaded cells in four randomly selected fields was determined. Manual counting of invaded cells in the lower chamber was performed in a representative area not located at the periphery.

### 4.11. Tube Formation Assay

Matrigel was dissolved at 4% overnight, coated onto 24-well plates at 150 µL per well, and solidified at 37 °C for 1 h. HTR8/SVneo cells were seeded at 1 × 10^5^ cells/well in serum-free RPMI 1640 medium. After 4 h of incubation, light microscopy images were captured from five random fields of each well at 100x magnification. Meshes were defined as areas enclosed by the segments. Manual counts of meshes were performed in five representative areas.

### 4.12. Statistical Analysis

The data are presented as the means ± standard deviations. Two-tailed Student’s *t* tests were used for comparisons between two groups. The statistical significance of the fold change in the transcript expression profile in the bioinformatics studies was determined using paired *t* tests. Statistical significance was set at *p* < 0.05, *p* < 0.01, and *p* < 0.001.

## 5. Conclusions

Our study identified differential AADAC expression between PE and normal placentas. In vitro assays further demonstrated that altered AADAC expression was associated with changes in trophoblast invasion and tube formation under hypoxia-mimicking conditions, processes known to be impaired in PE. These findings suggest that AADAC may participate in the functional regulation of trophoblasts relevant to placental dysfunction. However, given the exploratory nature of this study, further mechanistic and translational investigations are required to clarify the biological significance of AADAC and to assess its potential clinical utility.

## Figures and Tables

**Table 1:** Clinical characteristics of the study participants.

Characteristic	Normal (N = 15)	Preeclampsia (N = 30)	*p* Value
Maternal age (years)	34.9 ± 3.4	34.8 ± 4.1	0.957
Gestational age (weeks)	33.5 ± 5.0	30.9 ± 4.8	0.094
Prepregnancy maternal weight (kg)	58.8 ± 9.1	69.8 ± 16.8	0.007
Maternal weight at birth (kg)	68.4 ± 5.9	81.8 ± 16.9	<0.001
Prepregnancy BMI (kg/m^2^)	22.5 ± 4.0	26.7 ± 5.9	0.019
Systolic blood pressure (mmHg)	124.5 ± 10.9	173.0 ± 17.1	<0.001
Diastolic blood pressure (mmHg)	77.5 ± 9.7	107.8 ± 13.8	<0.001
Urine protein/creatinine ratio	0.1 ± 0.1	3.8 ± 4.9	<0.001

## Data Availability

The original contributions presented in this study are included in the article/Supplementary Material. Further inquiries can be directed to the corresponding author.

## Supplementary Materials

The following supporting information can be downloaded at: https://www.mdpi.com/article/10.3390/ijms27021103/s1.

## Abbreviations

The following abbreviations are used in this manuscript:

AADAC	Arylacetamide deacetylase
CoCl_2_	Cobalt chloride
CVD	Cardiovascular disease
EVT	Extravillous trophoblast
GO	Gene Ontology
HIF1-α	Hypoxia-inducible factor 1-alpha
mTOR	Mammalian target of rapamycin
NGS	Next-generation sequencing
OE	Overexpression
PE	Preeclampsia
PTM	Posttranslational modification
PI3K	Phosphatidylinositol 3-kinase
RNA	Ribonucleic acid
VSMCs	Vascular smooth muscle cells
