# Supplementary figures and images for: Altered AADAC Modulates Trophoblast Invasion and Suggests a Potential Angiogenic Regulatory Role in Severe Preeclampsia
Source: Int J Mol Sci. 2026 Jan 22;27(2):1103. doi: 10.3390/ijms27021103 (PMC12841916; doi:10.3390/ijms27021103)

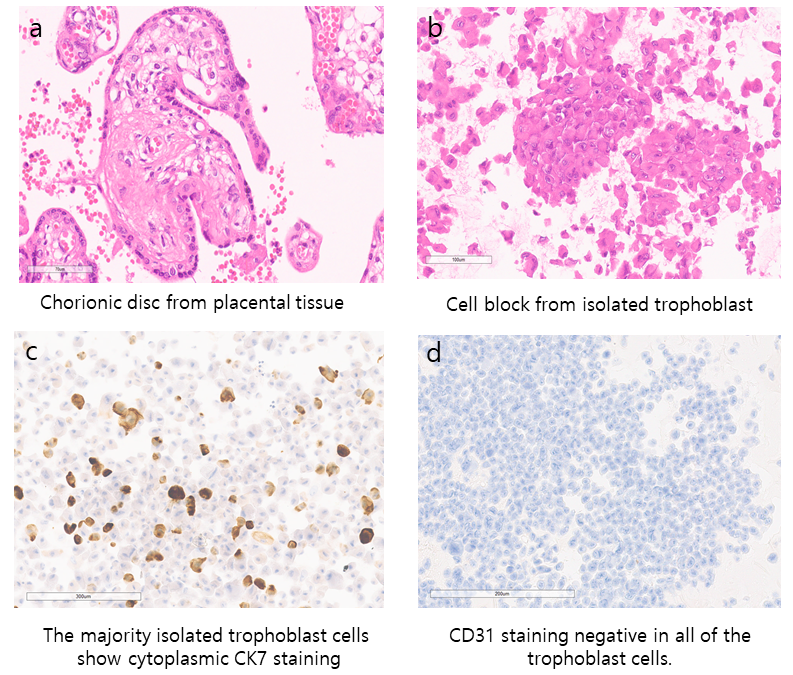

Supplement: Supplementary file 1 [file ijms-27-01103-s001.zip › figure S1.tif]

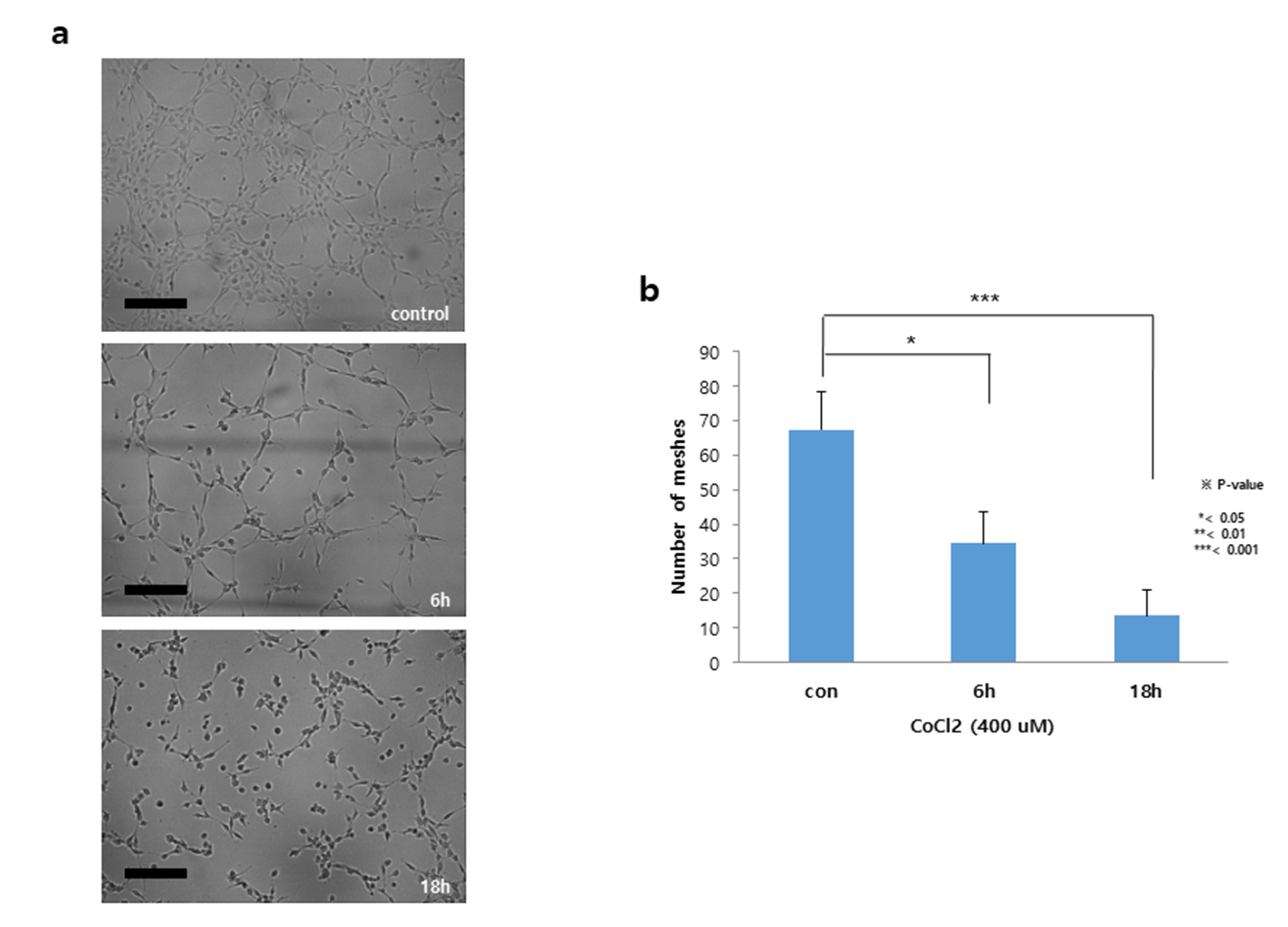

Supplement: Supplementary file 1 [file ijms-27-01103-s001.zip › figure S2.tif]

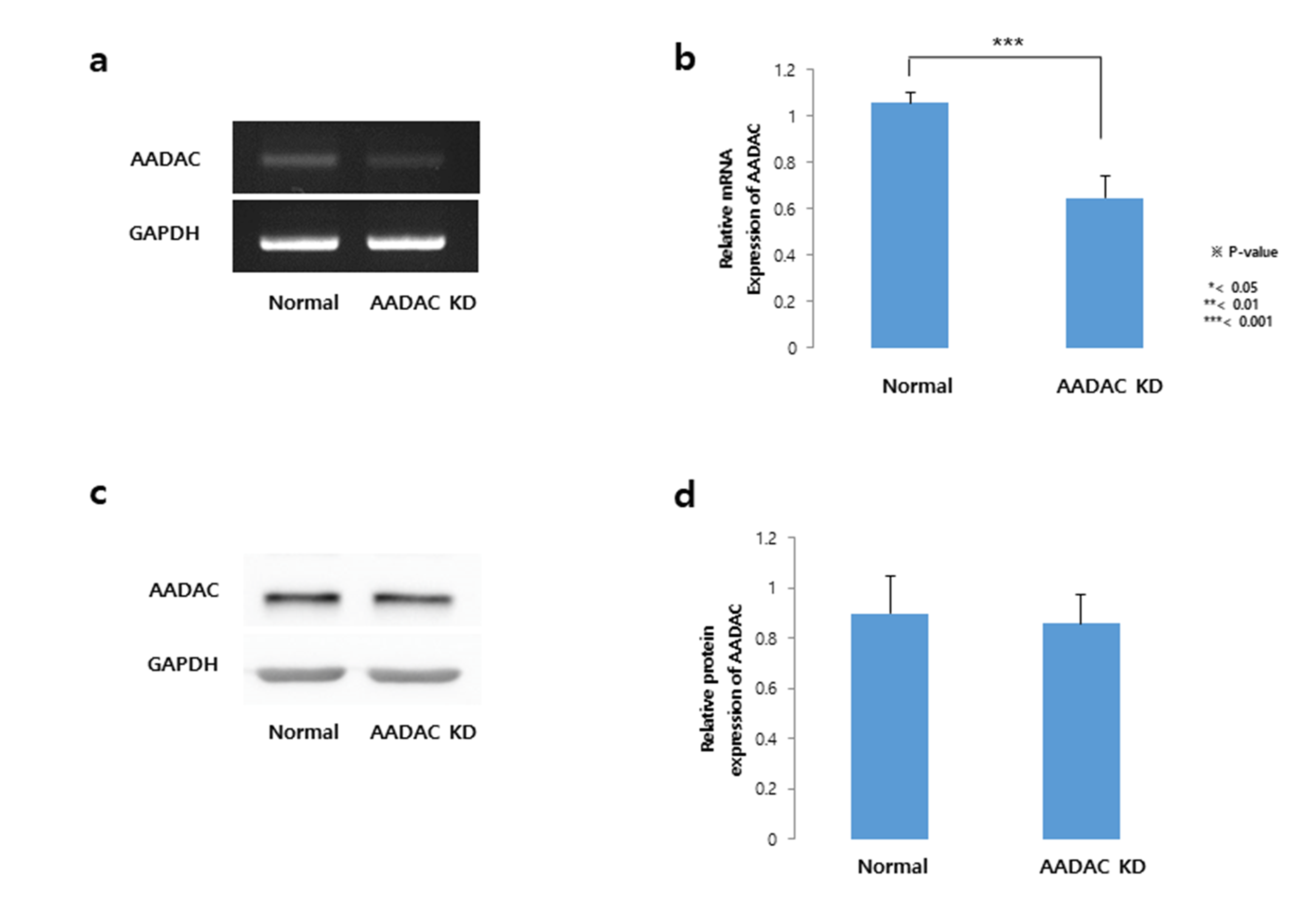

Supplement: Supplementary file 1 [file ijms-27-01103-s001.zip › figure S3.tif]

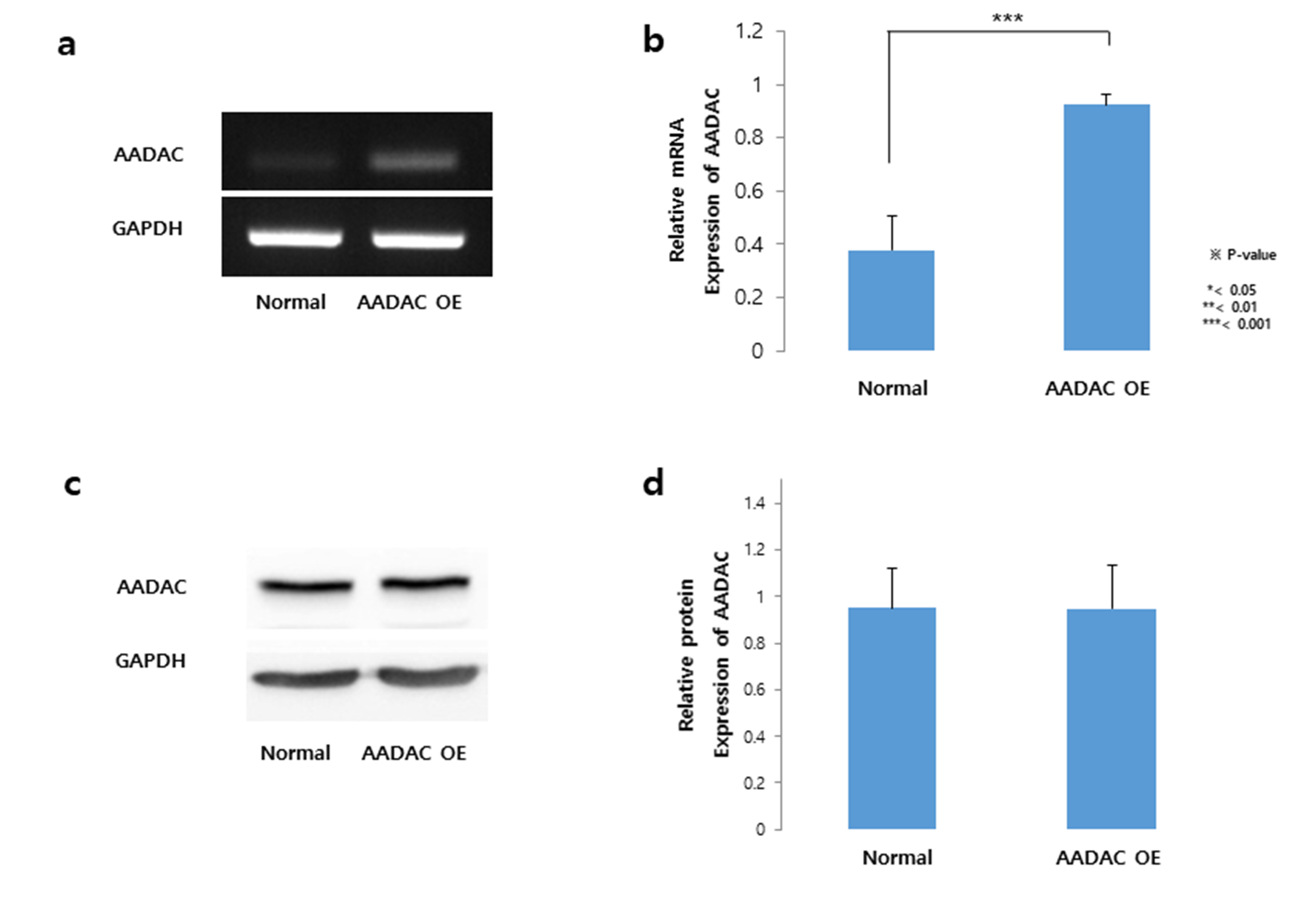

Supplement: Supplementary file 1 [file ijms-27-01103-s001.zip › figure S4.tif]
